# P01 Antibiotic efficacy treatment of community-acquired pneumonia among CMV-infected individuals

**DOI:** 10.1093/jacamr/dlad066.005

**Published:** 2023-06-14

**Authors:** Kiarina Chichirelo-Konstantynovych, Tetyana Konstantynovych

**Affiliations:** Department of Infectious Diseases, National Pirogov Memorial Medical University, Vinnytsya, Ukraine; Department of Propedeutics of Internal Medicine, National Pirogov Memorial Medical University, Vinnytsya, Ukraine

## Abstract

**Background:**

The investigation is devoted to possible antibiotic resistance formation and its adverse event prognostic influence among young patients with community-acquired pneumonia (CAP) with cytomegalovirus (CMV) persistence and absence of other comorbid pathology.

**Subjects and methods:**

The observation included 105 patients (average age 34.1 ± 0.8 years), which had been examined by epidemiological, clinical (including PORT prognostic scale) according to European Respiratory Society requirements, serological test for CMV IgM, IgG detection and antibiotic usage questionnaire for a 3-month period prior CAP.

**Results:**

76.2% (*n* = 80) CAP patients had positive CMV IgG. CMV-positive patients were characterized by higher severity outcome risk in the nearest future versus CMV-negative ones (*P* < 0.0001): third risk class—14.9 times higher, fourth risk class—10.5 times higher. CMV IgG–positive results correlated with the CAP severity level (*r* (Spearman) = 0.472). In total, 51.4% (*n* = 54, among them 47 CMV-positive patients) mentioned antibiotic use during the last 3 months prior to CAP. During the CAP treatment, 75.0% (*n* = 60) of CMV-positive patients and 20.0% (*n* = 5) of CMV-negative ones did not respond to antibiotic treatment by cephalosporin and quinolone lines and needed changes in aetiology prescription according to antibiotic susceptibility microbial test. 61.3% (*n* = 47) of CAP patients (everybody was after medication replacement) got adverse events like antibiotic-associated diarrhoea (16 cases), QT prolongation in ECG (17 cases), creatinine, urea elevation (11 cases) and alanine/aminotransferase elevation (5 cases).

**Conclusions:**

CMV persistence is associated with higher risk of severe CAP among young patients without comorbid pathologies and contributes to the antibiotic resistance creation as well as related side effects.

